# Psychological Empowerment on the Streets: Designing and Validating Multisensory Experiences in Simulated Autonomous Driving

**DOI:** 10.1111/nyas.70305

**Published:** 2026-06-16

**Authors:** Eunju Jeong, You Jeong Hong, Jiyeon Shin, Jong Su Kim, Moon A Yoo, Sung‐Phil Kim

**Affiliations:** ^1^ Department of Music Therapy, Graduate School Ewha Womans University Seoul South Korea; ^2^ Ewha Music Wellness Research Center Ewha Womans University Seoul South Korea; ^3^ Department of Intelligence and Information, Graduate School of Convergence Science and Technology Seoul National University Seoul South Korea; ^4^ Department of Industrial Engineering Seoul National University Seoul South Korea; ^5^ Department of Biomedical Engineering Ulsan National Institute of Science and Technology Ulsan South Korea

**Keywords:** autonomous driving, electroencephalography, multisensory stimulation, music, psychological empowerment, vibration

## Abstract

Driving is evolving from a transportation task into a rich, multisensory experience in automated vehicles. This study developed and evaluated three multisensory solutions combining music with synchronized vibrotactile stimulation for autonomous driving contexts: safe (city driving), engagement (highway cruising), and entertainment (highway entry). Eighteen healthy adult drivers experienced three context–solution pairs presented in music only (M) and music with vibration (MV) modalities with simulated autonomous driving scenarios. Participants’ responses were measured using self‐assessment manikin (SAM) ratings and electroencephalography (EEG). A significant main effect of modality showed that MV led to greater pleasure than M (EEG: *p* < 0.05; SAM: *p* < 0.05), with arousal showing a similar pattern (EEG: *p* < 0.05; SAM: *p* = 0.099). Behavioral data showed different emotional profiles across the three context−solution pairs (*p* < 0.001 for pleasure, arousal, and dominance), whereas the EEG contrast, which subtracted the video‐only condition, showed no significant pair effect. These findings demonstrate that vibrotactile enhancement provides consistent emotional benefits across diverse driving contexts. Because each musical solution was paired with a unique driving scenario, these differences cannot be attributed solely to the music intervention. Future optimization of music and vibrotactile parameters may further enhance the autonomous driving experience.

## Graphical abstract

This study developed and evaluated three multisensory solutions combining music with synchronized vibrotactile stimulation for autonomous driving contexts: safe (city driving), engagement (highway cruising), and entertainment (highway entry). Eighteen adults’ emotional responses were measured using self‐report and EEG. Adding vibration to music increased pleasure across all three pairs, with arousal showing a similar pattern. The three pairs produced different self‐reported emotional profiles. Our findings support adaptive multisensory design for in‐vehicle experiences.

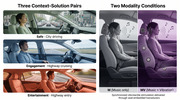

## 1 Introduction

Driving is not only a means of transportation, but a complex experience that shapes our daily lives and interactions. Initially, the primary focus was on ensuring the safety of all road users [1], which led to vehicle designs that supported the physical and cognitive functions of the drivers [2−3, 4]. Recent advancements in automated vehicle technologies introduced non‐driving‐related tasks such as sleeping, resting, watching YouTube, and gaming [5−6, 7, 8], and future mobility evolved into an integrated environment [9−10, 11]. Consequently, the focus shifted toward user‐centric solutions that align with psychological empowerment in these environments.

Psychological empowerment within a mobility ecosystem prioritizes safety, usability, and entertainment features, which correspond to strategies for enhancing technological acceptance [12, 13]. In‐vehicle empowerment begins with mitigating passenger anxiety and uncertainty [14, 15]. Achieving safety involves enhancing perception and sensing technologies [16] and implementing robust safety and security measures [17]. Further, control systems that ensure smooth operation [18] and effective path planning algorithms [19] contribute to usability by keeping passengers attentive yet relaxed and maintaining an optimized level of awareness and attention without any cognitive overload or additional commitment [20, 21]. Finally, entertainment features cater to passengers seeking excitement and stimulation. Integrating entertainment features such as games or interactive media can help enhance the in‐vehicle experience by making it more engaging and enjoyable [22−24].

These three experiential states can be conceptualized within Mehrabian and Russell's pleasure–arousal–dominance (PAD) model [25], a dimensional framework that maps emotional experiences across three independent axes: pleasure (pleasant to unpleasant), arousal (aroused to calm), and dominance (control to submission) [26]. Safety corresponds to high pleasure, low arousal, and moderate‐to‐high dominance, reflecting a calm yet secure state [27]. Engagement maps to neutral‐to‐positive pleasure, moderate arousal, and moderate‐to‐high dominance, maintaining optimal alertness without cognitive overload [28]. Entertainment is characterized by high pleasure, high arousal, and moderate dominance, providing stimulation and enjoyment within safe bounds [29].

Music has been recognized for its conscious and unconscious effects on affective modulation [30, 31] across the PAD dimensions [32, 33], making it particularly suitable for targeting specific PAD profiles in autonomous vehicle contexts. Furthermore, music can enhance feelings of safety, mindfulness, and excitement [34, 35], which are key aspects of psychological empowerment that can improve mood and emotions, as well as driving performance [36]. A growing body of literature has examined the influence of music on drivers’ emotional states and performance. Calm music has been shown to induce relaxation and alleviate driving‐related stress [37−39], although low‐intensity music may paradoxically impair vigilance [40]. Tempo manipulations have demonstrated that faster music increases arousal but may encourage risky driving behavior [41, 42], while researcher‐designed music backgrounds can enhance positive mood and safety [43]. The emotional valence and arousal level of music modulate physiological stress responses during driving [44], and driver‐selected music can maintain a positive mood and favorable driving behavior [45, 46]. More recently, Koch et al. [47] demonstrated that music‐based interventions delivered during daily commutes improved drivers’ well‐being. These findings establish that music can meaningfully modulate emotional states in driving contexts, though the direction and magnitude of effects depend on the specific musical parameters employed.

Beyond affective modulation through music, other studies have explored more direct applications of music in automated driving contexts, such as interactive drum‐playing systems [48] or extended‐reality systems in live music performances [23]. However, these approaches focused on entertainment without addressing safety requirements. This dual requirement becomes particularly important at SAE Level 3. As Miele et al. [49] noted, the automated system handles dynamic driving tasks, but drivers must remain receptive to takeover requests and ready to resume control within a limited time window. This conditional freedom, where drivers may temporarily engage in nondriving activities while maintaining takeover readiness, reduces workload but raises new design challenges for sustaining appropriate engagement levels. At higher automation levels, Nadri et al. [50] investigated auditory displays for Level 4 automated vehicles and found that audiovisual conditions significantly enhanced situation awareness during automation transitions. These findings suggest that in‐vehicle solutions must address both safety‐critical information and sustained passenger experience.

Although previous research demonstrated the potential of music to promote psychological empowerment in vehicular contexts, it has limitations in design specificity, generalizability, and ecological validity. Regarding design specificity, most studies have used pre‐existing or participant‐selected music rather than systematically designed stimuli targeting specific emotional profiles [40, 42, 43]. In terms of generalizability, auditory interventions in automated vehicles have primarily employed short, discrete signals rather than sustained music for continuous emotional modulation [51, 52]. Finally, these studies have focused on auditory stimulation only, and the potential of multisensory approaches, particularly the integration of music with vibrotactile feedback, has received less attention in automotive contexts.

Multisensory integration is the neural process by which information from different sensory modalities is combined to form a unified percept, and produces responses that exceed the sum of unisensory inputs [53]. The process is governed by the rule of temporal and spatial congruence and by the principle of inverse effectiveness [54]. Among sensory pairings, the auditory−tactile combination is particularly robust, as auditory and tactile perception share fundamental psychophysical properties [55]. Neurophysiological evidence indicates that auditory−tactile integration occurs at early stages of information processing and engages shared cortical regions [56−57, 58, 59]. Vibrations can be intuitively perceived and interpreted through touch, which is a fundamental human sensory channel, without adding a visual load; this makes them particularly suitable for seated vehicle occupants [60, 61]. Merchel and Altinsoy [62] demonstrated that integrating sound and vibration significantly improved the overall quality of the music experience. Haynes et al. [63] and Aker et al. [64] suggested that vibrations could enrich the music listening experience without disrupting the flow. Brodsky [65] further found that musicians exposed to music combined with vibrations reported deeper relaxation and more vivid imagery during imagery tasks.

Building on these findings, Siedenburg et al. [66] found that music‐congruent vibrotactile stimulation delivered through a chair significantly enhanced feelings of groove, the sense of being part of the music, and arousal. Multichannel vibrotactile presentation produced stronger enhancement than monorendering, suggesting that the spatial distribution of vibrotactile information contributes meaningfully to the multisensory experience. Schwartz et al. [67] further demonstrated that audio−tactile music integration enhanced positive emotions and reduced anxiety compared to unisensory auditory stimulation. The emotional effects of vibrotactile music enhancement are supported by neurophysiological evidence. García López et al. [68] found that vibrotactile stimulation activated brain regions involved in affective music processing, including the superior temporal gyrus, inferior frontal gyrus, and insula. Raheel et al. [69] demonstrated that tactile‐enhanced multimedia content elicited significantly different valence and arousal scores when the tactile modality was engaged.

In automotive contexts, vibrotactile feedback through the vehicle seat has been extensively studied for safety‐critical information delivery [4, 70]. The driver's seat provides an ideal platform for vibrotactile stimulation without adding visual or auditory load [71]. However, the application of seat‐based vibrotactile stimulation for emotional enhancement with continuous music has rarely been examined in vehicular environments. A recent scoping review [72] highlighted the potential for creative audio‐tactile mapping schemes, yet noted that rigorous evaluations of emotional effects in applied contexts are scarce. Consequently, investigating how continuous, music‐synchronized vibrotactile stimulation modulates emotional responses in autonomous driving contexts represents a critical gap. Unlike brief warning signals or event‐triggered haptic alerts, continuous vibrotactile accompaniment synchronized with music may enable more sustained modulation of affective states, making it relevant for highly automated environments where in‐cabin experience quality becomes a central design concern. Moreover, affective demands in automated driving are unlikely to be uniform across situations, raising the question of whether vibrotactile enhancement can provide a consistent emotional benefit across diverse driving contexts.

To address these gaps, the present study developed contextually adaptive, music‐based multisensory content integrating sustained background music with synchronized chair‐delivered vibrotactile stimulation for autonomous driving environments. Three context–solution pairs were designed: safe (city driving context with calm music solution), engagement (highway cruising context with steady music solution), and entertainment (highway entry context with energizing music solution). The primary aim was to examine whether the addition of vibrotactile stimulation to music (MV) enhances emotional responses compared to music alone (M). As a secondary, exploratory aim, we examined emotional profiles across the three context–solution pairs; however, because each musical solution was paired with a unique driving scenario, any observed differences cannot be attributed to the musical intervention alone, reflecting the combined influence of musical and contextual factors. Based on the multisensory integration literature and the PAD framework, we formulated the following hypotheses:

H1 (modality effects): The addition of synchronized vibrotactile stimulation (MV) will enhance emotional responses compared to music alone (M), as indicated by both self‐reported behavioral measures (pleasure, arousal, dominance) and neurophysiological electroencephalogram (EEG) indices (pleasure, arousal).
H2 (solution effects): The three context–solution pairs will show distinct emotional profiles.

## 2 Methods

### 2.1 Participants

A total of 18 healthy adults (*M* = 25.21 years, *SD* = 4.90) with driving experience (*M* = 3.47 years, *SD* = 2.66) voluntarily participated in the study. None of the participants had received professional musical training or had a history of neurological disorders or sensory impairments. This study employed a Latin square design with repeated measures to evaluate two independent variables: context−solution pairs (Safe, Engagement, Entertainment) and modalities (M, MV) on pre‐post changes in pleasure, arousal, and dominance. A Latin square design was selected to efficiently control order effects, minimize participant requirements, and systematically test multiple conditions without bias. A pre‐post design was employed to capture within‐session affective changes induced by each context−solution pair in both self‐reported responses and EEG measurements, following established protocols in music‐related research [32, 50, 73, 74]. Importantly, identical driving video content was presented across both M and MV conditions within each pair, thus any differences between modalities can be attributed to the auditory−tactile intervention rather than the driving scenario itself.

### 2.2 In‐Vehicle Multisensory System

#### 2.2.1 Music Design for Psychological Empowerment

We conceptualized the feelings of being safe, engaged, and entertained as the primary targets for our multisensory content, building on future mobility user experience and technology acceptance enhancement strategies. Based on the PAD framework [25, 26] and previous research mapping structural components of music to emotional dimensions [32, 33], we selected musical features that could systematically target these three emotional profiles. Juslin and Lindström [75] explored how certain musical features affect the emotional perceptions of the listeners. They determined that a combination of low pitch (below C4), minor mode, slow tempo (70 bpm), and uneven rhythm with a long‐to‐short ratio of 3:1 effectively conveys tenderness. Simple melodic progression, legato articulation, soft timbre (with a higher proportion of high‐frequency energy), and low sound level were identified as significant components that induced emotions with low arousal and positive valence. Similarly, Eerola et al. [76] found that the mode was the most influential component for inducing certain types of emotion. Tempo, register, dynamics, articulation, and timbre followed; however, their ranking varied among the different emotions. For emotions characterized by positive valence and low arousal (e.g., peacefulness), a combination of major and minor modes, slow tempo (1.2 notes per second), middle register (around a MIDI note number of 65), minimal dynamics, and woodwind timbre were identified as effective components. For tempo and loudness, Schubert [77] used a continuous response methodology for investigating the relationship between musical features and perceived emotions. Changes in tempo and loudness are associated with changes in arousal. McAdams et al. [78] found that certain features of timbre, along with pitch, can elicit different types of emotions.

**FIGURE 1:**
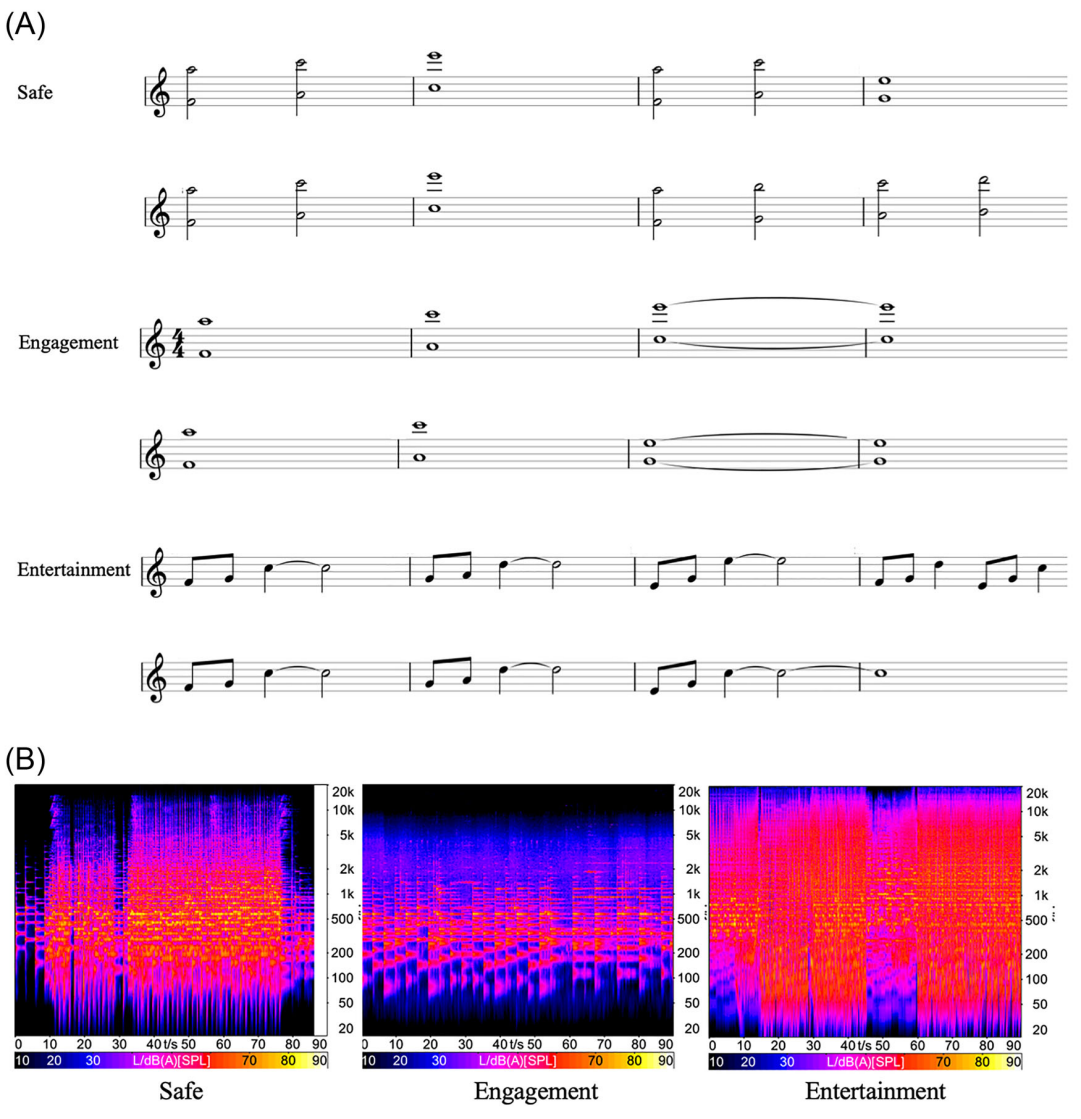
Example of (A) scores and (B) spectrograms of the A‐weighted sound pressure levels for music pieces composed to reflect Safe, Engagement, and Entertainment.

Based on these previous findings, we designed music stimuli using the same theme and harmonic structure, so that the three types of music content maintained some level of consistency. For Safe music, we used a binary form structure, with a tonal range predominantly in the alto‐tenor register. The tempo was set at a moderate pace of 70–80 beats per minute (BPM), with a dynamic mezzo‐forte level, which indicates a medium level of musical intensity. For Engagement music, a tonal range in the tenor‐soprano register and a steady tempo of 60–70 BPM were used to provide a calm and steady rhythmic foundation. The dynamics vary from piano to mezzo forte, aiming to create a soft yet engaging sound level. Finally, for Entertainment music, a faster tempo of 112–122 BPM and a dynamic range from mezzo‐forte to forte ensure a lively and vibrant atmosphere. All musical pieces were composed using Logic Pro X (Apple Inc., USA) by the first and third authors. Figure 1 shows the samples of score and spectrogram of the composed music corresponding to each of the three musical concepts.

#### 2.2.2 Design of the Vibrotactile System

A chair‐type multisensory interface was developed to provide a cohesive and immersive experience, integrating musical and vibrotactile stimuli [79]. The system architecture consisted of three main components: audio signal processing, music‐to‐vibration mapping, and vibrotactile transduction. For audio signal processing, we utilized a Focusrite Scarlett 18i20 (Focusrite Audio Engineering Ltd., UK) audio interface to minimize signal distortion (48 kHz sampling rate, 24‐bit resolution). Audio signals were analyzed in real‐time to extract energy parameters for vibration intensity control. The overall system latency was maintained at 13.7 ms through optimized I/O buffering (512 samples), well below the human perceptual threshold of 20–30 ms for audiovisual−tactile integration.

For music to vibration mapping, music sources were separated by instrument based on their functional roles within the musical structure and assigned to specific transducer locations. Our system employed a role‐based mapping informed by musical hierarchy, which aligns with established methods in music‐to‐vibration conversion research, including timbre‐based instrument separation and frequency‐to‐body region mapping [55, 80, 81, 82]. The top transducers, positioned in the backrest upper region and responsive to frequencies of approximately 277 Hz and above, were assigned to melodic components. The middle transducers, located in the backrest middle region and covering 100–500 Hz, were assigned to harmonic components. The base transducers, embedded in the seat cushion in a quadruple configuration and operating at 15–100 Hz, were assigned to rhythmic patterns.

**FIGURE 2:**
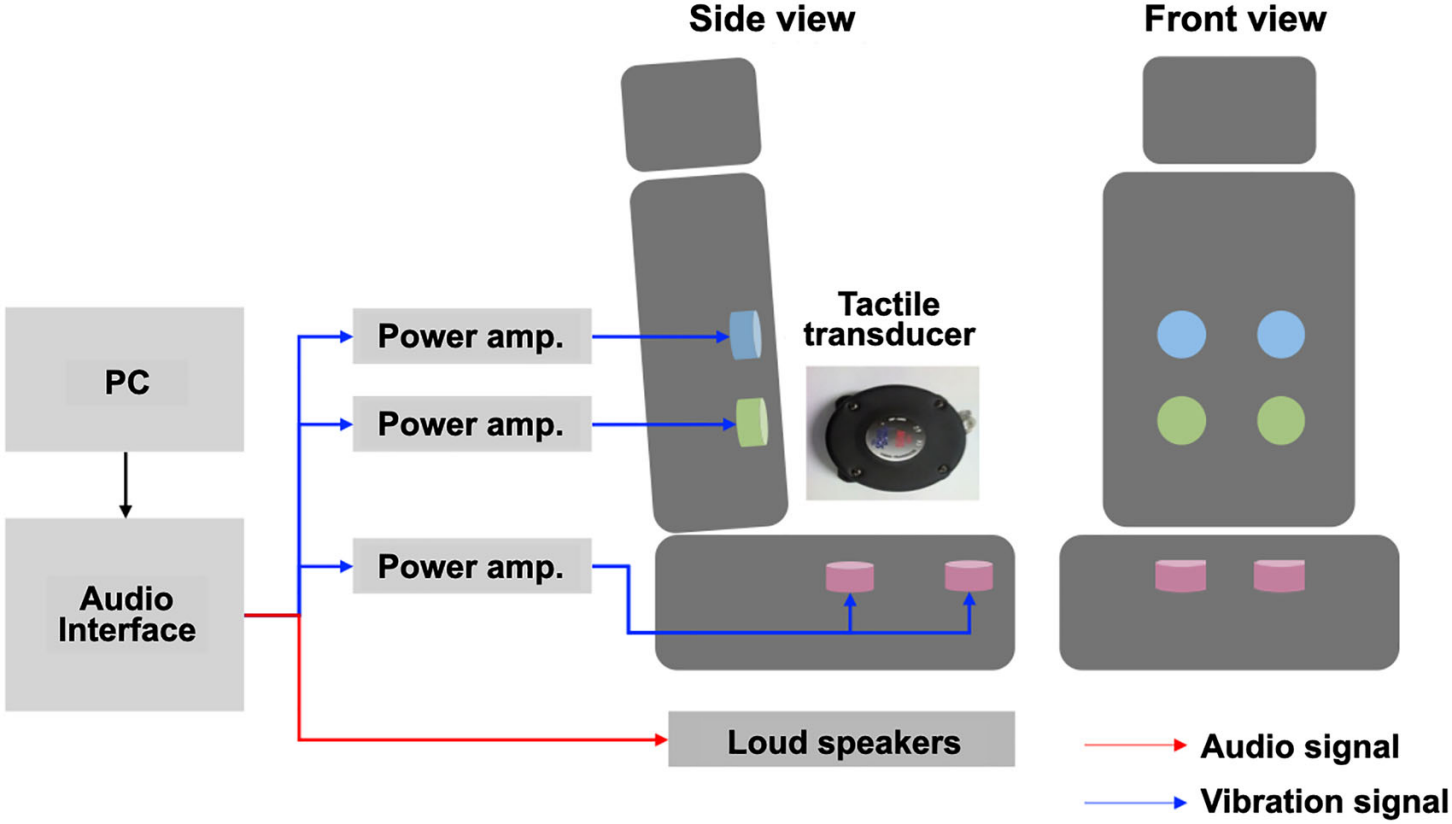
Schematic of the system developed to transmit music‐induced vibrations through the car seat.

Each separated audio channel from the interface was transmitted to an independent high‐output power amplifier (Kinter MA‐180, Kinter Audio, Shenzhen, China), providing a stable DC voltage (12–13 V). The amplifiers were impedance‐matched to the tactile transducers: 4Ω for the base transducers to optimize low‐frequency energy transmission, and 8Ω for the top and middle transducers for nuanced expression. Eight tactile transducers (SoundPower, Seoul, Republic of Korea) were embedded in the chair: four in the backrest and four in the seat cushion. All transducers were installed in custom‐carved foam pockets approximately 2 inches (5 cm) deep to prevent mechanical noise transmission to the chair frame and to focus vibratory energy exclusively toward the participants. Vibration intensity was dynamically modulated in real‐time based on magnitude analysis of the music signal, allowing vibratory energy to change organically in response to musical events (e.g., crescendos, climaxes) to maximize stimulus naturalness and immersion. Additionally, the system complied with ISO 2631 and EU Directive 2002/44/EC standards for whole‐body vibration exposure (weighted acceleration <0.3 m/s^2^). Transducer positioning and intensity were optimized based on automotive seating posture research [83] and whole‐body frequency range research [84−85, 86] to align with ergonomic best practices. A schematic of the system architecture is shown in Figure 2.

#### 2.2.3 Driving Scenario

We designed three primary driving scenarios based on previous research identifying key needs in automated vehicles [12, 13]. We specified a driving journey map through focus group interviews with four experienced drivers (mean age of 26 years; mean driving experience of 4.75 years), then refined using the standard driving network framework established by Korea Expressway Corporation (2022) [87] and previous studies that employed complex and boundary driving scenarios [88−89, 90]. Each music stimulus was paired with a specific driving scenario (Safe music for city driving, Entertainment music for highway entry, Engagement music for highway cruising) to ensure ecological validity. This design reflects real‐world applications where specific in‐vehicle interventions are deployed in their intended contexts rather than across all driving situations, allowing us to test whether multisensory solutions function effectively in their intended use cases. Additionally, this design enabled efficient counterbalancing of scenario−solution pairings across participants while keeping the total session duration sufficiently brief to ensure reliable EEG measurement. The sensory modality (multisensory vs. music‐only) for each solution was counterbalanced across sets.

The complete driving sequence consisted of five phases: (1) departure from a stationary parking position and initial acceleration toward urban roads; (2) city driving through congested urban networks; (3) highway entry and acceleration via on‐ramp merging onto the high‐speed traffic; (4) highway cruising at stable speed (∼100 km/h); and (5) highway exit through toll plaza deceleration. The three types of music (Safe, Entertainment, Engagement) were each paired with one specific driving scenario (city, highway entry, highway cruising, respectively). City driving scenario depicted complex urban driving conditions, including congested roads, lane changes, vehicles merging into traffic, and minor near‐collision events. These stressful urban elements create high cognitive load and negative affect, providing an ecologically valid context. The highway entry and acceleration scenario simulated the dynamic transition from urban roads to high‐speed highway traffic via an on‐ramp. The acceleration phase and merging maneuvers naturally elicit moderate‐to‐high arousal. The highway cruising scenario depicted sustained highway driving at a consistent cruising speed (∼100 km/h) after successful takeover. The monotonous, low‐variability environment is prone to inducing driver drowsiness and disengagement. Table S1 presents an overview of driving scenarios, phases, and solutions.

### 2.3 Measures

#### 2.3.1 Self‐Assessment Manikin

The emotional experiences of the participants were evaluated using self‐reported measures of pleasure, arousal, and dominance, assessed using the self‐assessment manikin (SAM) [91, 92]. Pleasure was conceptualized as the extent to which an individual experienced joy, psychological comfort, or satisfaction, while arousal was defined as the level of excitement or activation. Dominance refers to the perceived sense of control and autonomy. Each dimension was measured on a 7‐point Likert scale supplemented with illustrative visual representations to enhance clarity and facilitate accurate participant responses.

#### 2.3.2 Electroencephalography

EEG signals were recorded using a wireless EEG headset (DSI‐24; Wearable Sensing, USA), which included 21 active dry electrodes positioned based on the international 10–20 system (Fp1, Fp2, Fz, F3, F4, F7, F8, Cz, C3, C4, T7/T3, T8/T4, Pz, P3, P4, P7/T5, P8/T6, O1, O2, A1, and A2). Electrodes A1 and A2, located on the left and right earlobes, respectively, served as reference electrodes during data acquisition. The EEG data were sampled at a rate of 300 Hz. Prior to initiating the experiment, the signal stability and electrode contact quality were verified using the DSI‐Streamer software (Wearable Sensing) to ensure reliable data collection, which enabled us to confirm adequate contact between the dry electrode sensors and the scalp. The signal amplitude was maintained within a range of ±100 µV. EEG was selected as the primary measure since galvanic skin response is highly susceptible to confounds from vibrotactile stimulation, which was a core component of our experimental design. Tactile stimulation can directly trigger electrodermal responses through somatosensory activation independent of affective arousal [93], making it difficult to isolate emotion‐related arousal from stimulus‐driven skin conductance changes. Second, EEG enables simultaneous assessment of both affective dimensions (valence and arousal) through distinct neural signatures, allowing for integrated examination of affective states within a single modality [94, 95].

### 2.4 Procedure

This study was approved by the Institutional Review Board of Hanyang University (HYUIRB‐202204‐003). Participants were recruited voluntarily through advertisements on the university website from April 8 to July 30, 2022. Upon arrival at the experimental sites, the participants were briefed on the research purpose and procedures according to the Declaration of Helsinki. The participants were required to sign a consent form before participating in the study. Participants first received detailed explanations of the experimental procedures, and subsequently, they were seated in a car seat equipped with a vibration generator at the back. Then, a dry EEG headset was applied. The EEG signal quality and stability were verified before the start of the experiment. The experiment was commenced only after signal stability was confirmed. Before beginning the main experiment, participants were presented with a set of international affective picture system (IAPS) [96] stimuli and asked to rate their emotional responses using the SAM. After their responses, the average values for the IAPS stimulus from the official IAPS database were displayed to help participants better understand the SAM rating process. This step was conducted prior to the main experiment for maximizing familiarity and accuracy using the SAM.

The participants were then randomly assigned to one of three experimental sets, each presenting the same three driving scenarios (city driving, highway entry, highway cruising) in identical order but with different solution‐scenario pairings. Within each set, one scenario was paired with MV, another with the same solution in M, and the remaining scenario was presented without a solution. The complete driving journey consisted of five phases. Phase 1 began with a 60‐s baseline capturing startup and initial urban movement. Phase 2 consisted of a 60‐s urban stress induction segment featuring lane changes, vehicles merging into traffic, and minor near‐collision events, followed by the Safe solution during a subsequent 90 s to mitigate negative affect and stabilize driver state. Phase 3 simulated a 60‐s transition from urban roads to high‐speed traffic via an on‐ramp, with the Entertainment solution tested during a subsequent 90 s to enhance the excitement associated with highway entry. Phase 4 depicted sustained cruising at approximately 100 km/h, introducing the Engagement solution during a 90‐s cruising phase to counter the drowsiness and disengagement typical of monotonous driving. Phase 5 concluded with a 60‐s highway exit and toll plaza deceleration, returning to the starting point. Following each driving scenario, the corresponding video continued to allow uninterrupted measurement of physiological and subjective responses during pre‐ and post‐solution phases: city driving transitioned to stable urban flow without lane changes or collision events, highway entry shifted to consistent speed without further acceleration, and highway cruising maintained steady speed throughout.

**FIGURE 3:**
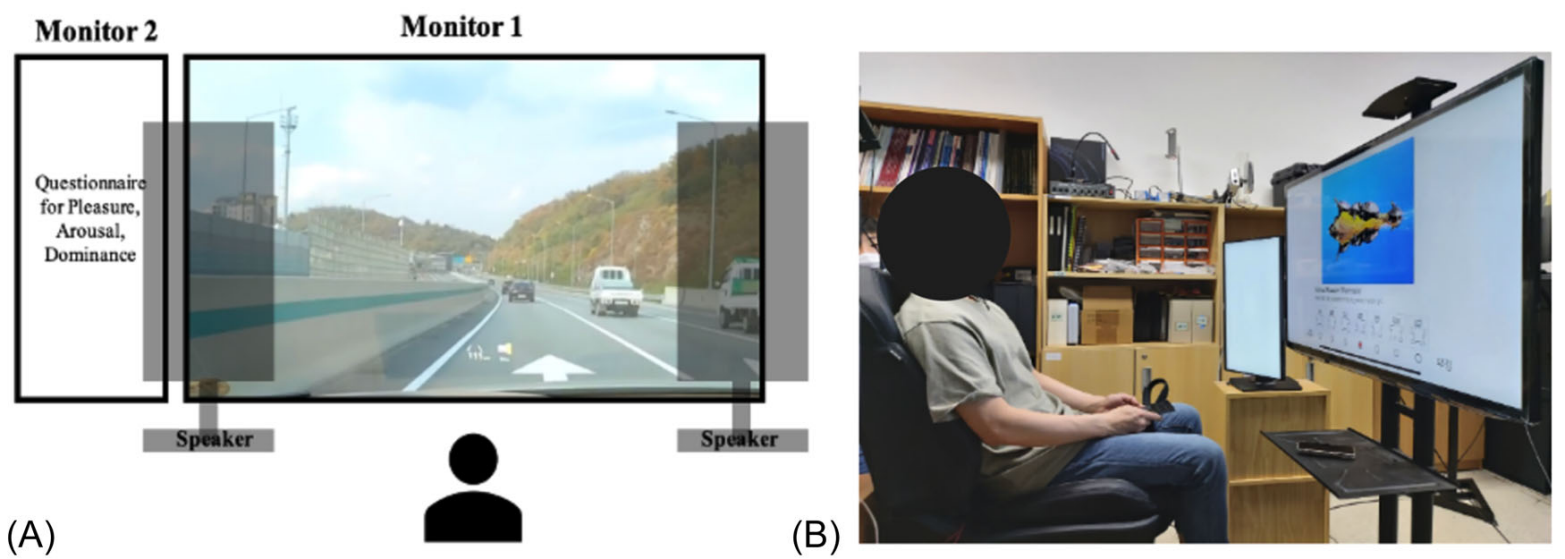
Example of experimental setting: (A) graphical illustration and (B) actual environment.

The experimental setup consisted of a 46‐inch primary monitor positioned at the participant's eye level, displaying dashboard‐camera video recording filmed from a driver's perspective on actual Korean roads, with a secondary monitor placed to the left for self‐report assessments (Figure 3). To create an immersive environment, each scenario integrated ambient driving sounds (engine noise, road surface friction, surrounding traffic, wind noise) mixed as a separate audio track, along with vibrotactile feedback derived from real‐time magnitude analysis of the driving audio. Music was presented through a pair of Focal Alpha 65 active studio monitors (Focal‐JMlab, Saint‐Étienne, France) positioned in an equilateral triangle configuration (60° angle, approximately 1 m distance) at ear height, adhering to ITU‐R BS.775 standards for stereo imaging and calibrated in a low‐noise environment (44–46 dB SPL background). Participants rated levels of valence, arousal, and dominance on the secondary monitor both before and after exposure to each solution; video‐only without solution were not evaluated as the primary comparison focused on multisensory versus music‐only modalities. EEG data were continuously recorded throughout each set. The time required to complete each set was approximately 15 min.

### 2.5 EEG Preprocessing and Analysis

EEG preprocessing was conducted in MATLAB using the EEGLAB toolbox. Raw EEG data were filtered using a bandpass filter (1–50 Hz) to remove low‐frequency drift and high‐frequency noise. Bad channels were automatically detected based on excessively high‐frequency noise (z‐scored electromagnetic noise‐to‐signal ratio) and low correlation with random sample consensus based spatial reconstructions across sliding windows. The detected bad channels were subsequently interpolated using spherical interpolation. The data were referenced to the common average reference. Artifacts were removed using an adaptive mixture‐independent component analysis algorithm. Independent components representing artifacts such as eye movements or muscle activity were excluded based on spatial patterns, classification probabilities from the ICLabel, and dipole fitting results. The preprocessed EEG signals were decomposed into several canonical frequency bands for extracting the EEG indices for emotions: theta (4–8 Hz), alpha (8–12 Hz), and high‐beta (25–38 Hz). The band‐specific power was computed by applying the Hilbert transform to the filtered signals, which yielded an instantaneous amplitude. The squared magnitude of the amplitude envelope was used to estimate the power of each frequency band.

Frontal alpha asymmetry (FAA) was used for computing the EEG index for emotional pleasure. FAA is widely known as a key neural marker associated with emotional and motivational processes [97]. Thus far, numerous studies have linked FAA to positive and negative valence, which plays a crucial role in shaping emotional experiences [98, 99]. In the present study, we defined FAA as the ratio of the average alpha power at Fp2 and F4 to that at Fp1 and F3. The index was subsequently computed as the difference between right‐ and left‐hemisphere alpha power values in the logarithmic (dB) scale.

**FIGURE 4:**
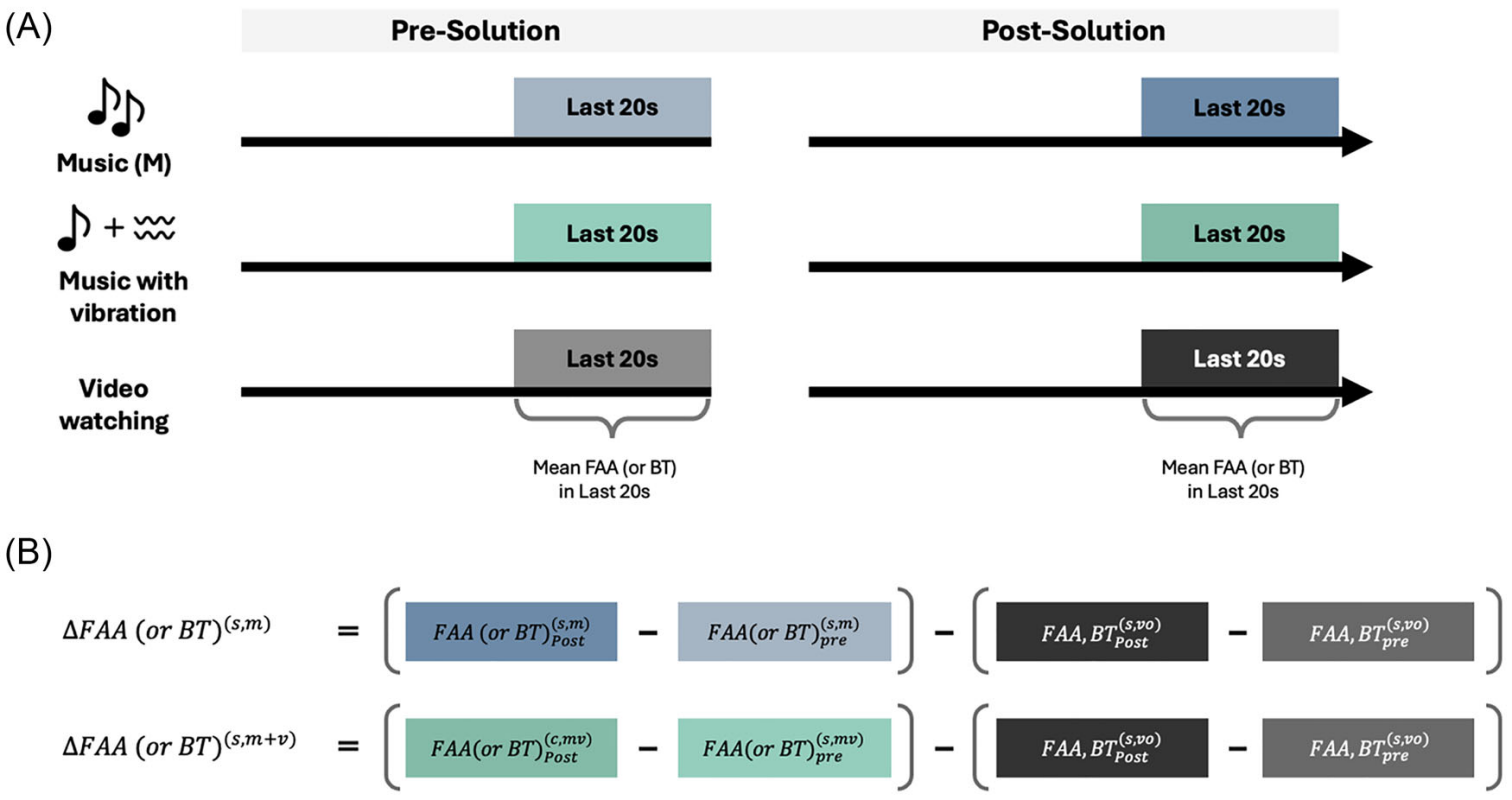
Schematic of the EEG index calculation procedure across experimental conditions and segments. (A) Segmentation and windowing strategy. EEG data for each trial were divided into two main segments: pre‐solution (baseline period prior to stimulus exposure) and peri‐solution (period during exposure to the solution). For each segment, the mean frontal alpha asymmetry (FAA) and high‐beta/theta power ratio (BT) were calculated within the last 20‐s windows across three conditions: music‐only, music + vibration, and control. (B) Contrast calculation equations. To isolate solution‐induced effects, a double subtraction approach was employed, as shown in the equations.

To evaluate stimulus‐induced affective shifts, each experimental trial was segmented into a pre‐solution and post‐solution period. For each segment, FAA values were extracted from the final 20 s (Figure 4A), which was selected to ensure signal stability and to capture the period during which affective responses were expected to be reliably observable. Within each experimental condition, pleasure‐related effects were quantified as the change in FAA from the pre‐solution to post‐solution segments, computed as the mean FAA values extracted from the final 20 s of each segment. These within‐solution FAA changes induced by each modality (M, MV) were then contrasted against the corresponding changes observed in the video‐only, without‐solution section (VO). Specifically, for each solution (s∈{safe,engagement,entertainment}) and modality (m∈{music,music+vibration}), the FAA contrast was computed as:

(1) $$\begin{array}{ccc} & & \Delta \;\;FAA^{\left(s,\;m\right)}=\\ & & \left(FAA_{post}^{\left(s,\;m\right)}-\;FAA_{pre}^{\left(s,\;m\right)}\right)\;-\;\left(FAA_{post}^{\left(s,\;vo\right)}-\;FAA_{pre}^{\left(s,\;vo\right)}\right), \end{array}$$

where $\Delta \;FAA^{\left(s,\;m\right)}$ denotes the modality‐specific change in FAA relative to the VO within the solution *s*. As shown in Figure 4B, this computation was designed to provide a robust measure of solution‐induced neural responses, where the control condition serves as a reference.

The EEG‐based index for arousal was obtained by averaging the high‐beta/theta power ratio (BT) across three frontal electrodes (Fz, F3, and F4). Previous studies showed that theta oscillations are modulated by emotional status, which has been assessed using arousal‐related facial expressions and emotional pictures [100, 101]. Using the same framework as in FAA, arousal‐related effects were quantified by computing within‐solution changes in BT between the pre‐solution and post‐solution segments and contrasting these changes against the VO (Figure 4B). In addition, beta‐band activity has been implicated in emotion‐related neural dynamics, with connectivity and coherence analyses demonstrating enhanced beta‐band involvement across multiple emotional states [102]. For each solution (*s*) and modality (*m*), the contrast of BT changes $\Delta \;BT^{\left(s,\;m\right)}$ was given by:

(2) $$\Delta \;\;BT^{\left(s,\;m\right)}=\left(BT_{post}^{\left(s,\;m\right)}-\;BT_{pre}^{\left(s,\;m\right)}\right)\;-\;\left(BT_{post}^{\left(s,\;vo\right)}-\;BT_{pre}^{\left(s,\;vo\right)}\right).$$

Although dominance has been proposed as a potential measure for assessing emotions along with arousal and pleasure, it is yet to be widely employed in EEG‐based emotion research due to the lack of well‐established neural indices. Accordingly, dominance was not included in the present study.

### 2.6 Statistical Analysis

#### 2.6.1 Behavioral Analysis

Self‐reported emotional experience data collected using the SAM questionnaire (pleasure, arousal, dominance) were rescaled from the original range of −3 to +3 to a range of 1–7 to ensure all values remained positive. To quantify the emotional shifts elicited by each of the experimental stimuli, delta values (Δ) were calculated for each of the pleasure, arousal, and dominance dimensions. These values represent the change in emotional state, derived by subtracting the pre‐ from the post‐solution ratings (i.e., Post‐Pre). This calculation was performed across all six experimental conditions, defined by the crossing of Modality (M, MV) and context–solution pair (Safe, Entertainment, Engagement), which was included as an exploratory factor to characterize emotional profiles. By utilizing these delta scores, the analysis focuses on comparing the relative impact of each content condition on the participants’ emotional transitions.

Given the non‐normal data distribution indicated by the Shapiro–Wilk test, relatively small sample size, and presence of two within‐subject factors, statistical analyses were conducted using the aligned rank transform (ART). ART, which is a nonparametric method designed for factorial design, enables examining both main and interaction effects while maintaining rank‐based properties inherent to nonparametric data [103−104, 105]. This approach was selected as an alternative to traditional parametric methods such as ANOVA, which require assumptions of normality that were not met in the present data. Where applicable, post‐hoc pairwise comparisons were conducted using Holm's correction for adjusting multiple comparisons, with the statistical significance set at *p* < 0.05. Statistical analyses of the behavioral data were performed using R (version 4.5.1).

#### 2.6.2 EEG Analysis

For EEG data, statistical analyses were performed separately for pleasure‐ and arousal‐related EEG indices. For each index, a two‐way repeated‐measures analysis of variance (rmANOVA) was performed with Modality (M, MV) and context–solution pair (Safe, Engagement, Entertainment) on the changes between pre‐ and post‐solutions. Prior to conducting the rmANOVA, the assumptions of normality and sphericity were tested. Normality was assessed using the Kolmogorov−Smirnov test, and Mauchly's test was applied to evaluate the sphericity. If the assumption of sphericity was violated, the Greenhouse−Geisser correction was applied. Where significant effects were found, Bonferroni‐corrected post‐hoc tests were performed to identify pairwise differences, with the significance level set at *p*< 0.05. Statistical analyses of the EEG data were performed using MATLAB.

## 3 Results

### 3.1 Behavioral Findings

#### 3.1.1 Pleasure

**FIGURE 5:**
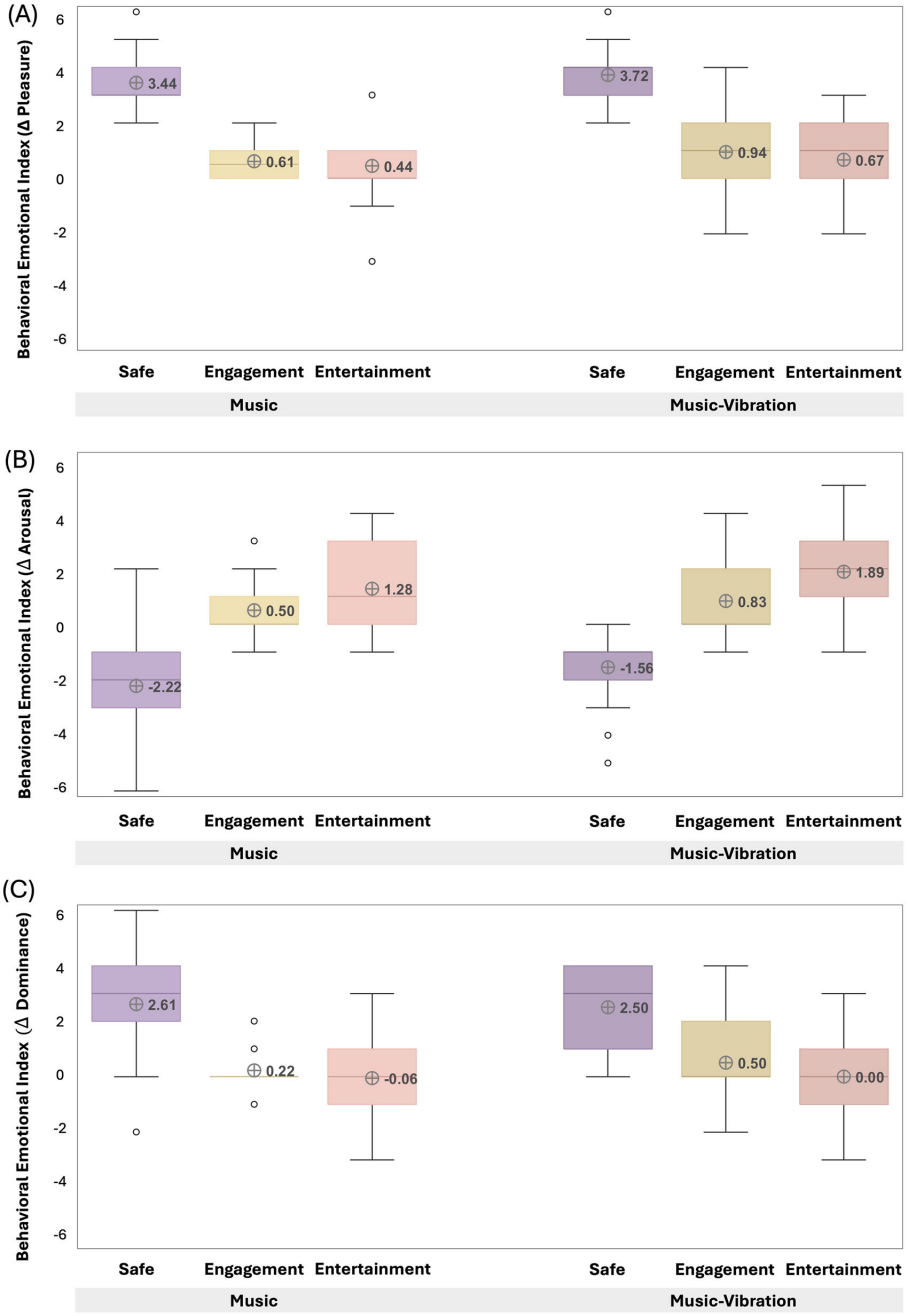
Condition‐wise comparison on pleasure, arousal, and dominance dimensions (behavior). Boxplots illustrate the differences in (A) pleasure, (B) arousal, and (C) dominance indices across context−solution pairs (Safe, Engagement, and Entertainment), and modality (M vs. MV). Each box represents the interquartile range (IQR), the central line indicates the median, “o” denotes outliers beyond 1.5× IQR, and “⊕” represents the mean value.

Descriptive statistics are detailed by context–solution pairs and modalities in Table 1 and Figure 5. A significant main effect of modality was observed (*F*(1,85) = 4.30, *p* < 0.05), where the MV condition (Δ (Post‐Pre Mean) = 1.778) led to a greater increase in pleasure than the M condition (Δ (Post‐Pre Mean) = 1.500). A significant main effect of context–solution pair was also observed (*F*(2,85) = 102.46, *p* < 0.001). Post‐hoc analyses revealed that the Safe context–solution pair showed a significantly greater increase in pleasantness (Δ (Post‐Pre Mean) = 3.583) compared to both the Engagement pair (Δ (Post‐Pre Mean) = 0.778; *t*(85) = 11.81, *p* < 0.001) and Entertainment pair (Δ (Post‐Pre Mean) = 0.556; *t*(85) = 12.91, *p* < 0.001). No significant difference was found between the Engagement and Entertainment pairs (*t*(85) = 1.10, n/s). The interaction effect between context–solution pair and modality was not statistically significant (*F*(2,85) = 0.02, n/s).

**TABLE 1:** Descriptive statistics of self‐reported measures (Pleasure, Arousal, and Dominance) by context−solution pairs (Safe, Engagement, and Entertainment) and modality (M, MV).

Emotion profile		Safe		Engagement		Entertainment	
		M	M+V	M	M+V	M	M+V
Pleasure	Mean	3.444	3.722	0.611	0.944	0.444	0.667
	Median	3	4	0.5	1	0	1
	SD	1.199	1.227	0.698	1.434	1.542	1.328
	IQR	1	1	1	1.5	1	1.75
Arousal	Mean	−2.222	−1.556	0.5	0.833	1.278	1.889
	Median	−2	−1	0	0	1	2
	SD	1.957	1.423	1.098	1.581	1.602	1.568
	IQR	1.75	1	1	2	3	2
Dominance	Mean	2.611	2.5	0.222	0.5	−0.056	0
	Median	3	3	0	0	0	0
	SD	1.944	1.465	0.808	1.689	1.349	1.534
	IQR	2	3	0	1.75	1.75	1.5

*Note*: M refers to music, and M+V refers to music with vibration conditions.

#### 3.1.2 Arousal

The main effect of modality was marginally significant (*F*(1,85) = 2.79, *p* = 0.0987), suggesting a potential trend toward higher arousal in the MV condition (Δ (Post‐Pre Mean) = 0.389) compared to the M condition (Δ (Post‐Pre Mean) = −0.148). A significant main effect of context–solution pair was found (*F*(2,85) = 65.18, *p* < 0.001). The highest arousal increase was observed in the Entertainment context–solution pair (Δ (Post‐Pre Mean) = 1.583), which was significantly higher than the Engagement pair (Δ (Post‐Pre Mean) = 0.667; *t*(85) = −2.94, *p* < 0.01) and Safe pair (Δ (Post‐Pre Mean) = −1.889; *t*(85) = −11.02, *p* < 0.001). The Engagement pair also elicited significantly higher arousal than the Safe pair (*t*(85) = −8.08, *p* < 0.001). No significant interaction between context–solution pair and modality was found (*F*(2,85) = 0.11, n/s).

#### 3.1.3 Dominance

Neither the main effect of modality (*F*(1,85) = 0.20, n/s; Δ (Post‐Pre Mean) in M = 0.926; Δ (Post‐Pre Mean) in MV = 1.000) nor the interaction between context–solution pair and modality (*F*(2,85) = 0.23, n/s) reached statistical significance. A significant main effect of context–solution pair was observed (*F*(2,85) = 34.34, *p* < 0.001). The Safe context–solution pair showed a significantly greater increase in dominance (Δ (Post‐Pre Mean) = 2.556) compared to both the Engagement pair (Δ (Post‐Pre Mean) = 0.361; *t*(85) = 6.25, *p* < 0.001) and the Entertainment pair (Δ (Post‐Pre Mean) = −0.028; *t*(85) = 7.84, *p* < 0.001). No significant difference was found between the Engagement and Entertainment pairs (*t*(85) = 1.59, n/s).

### 3.2 EEG Findings

#### 3.2.1 Pleasure

**FIGURE 6:**
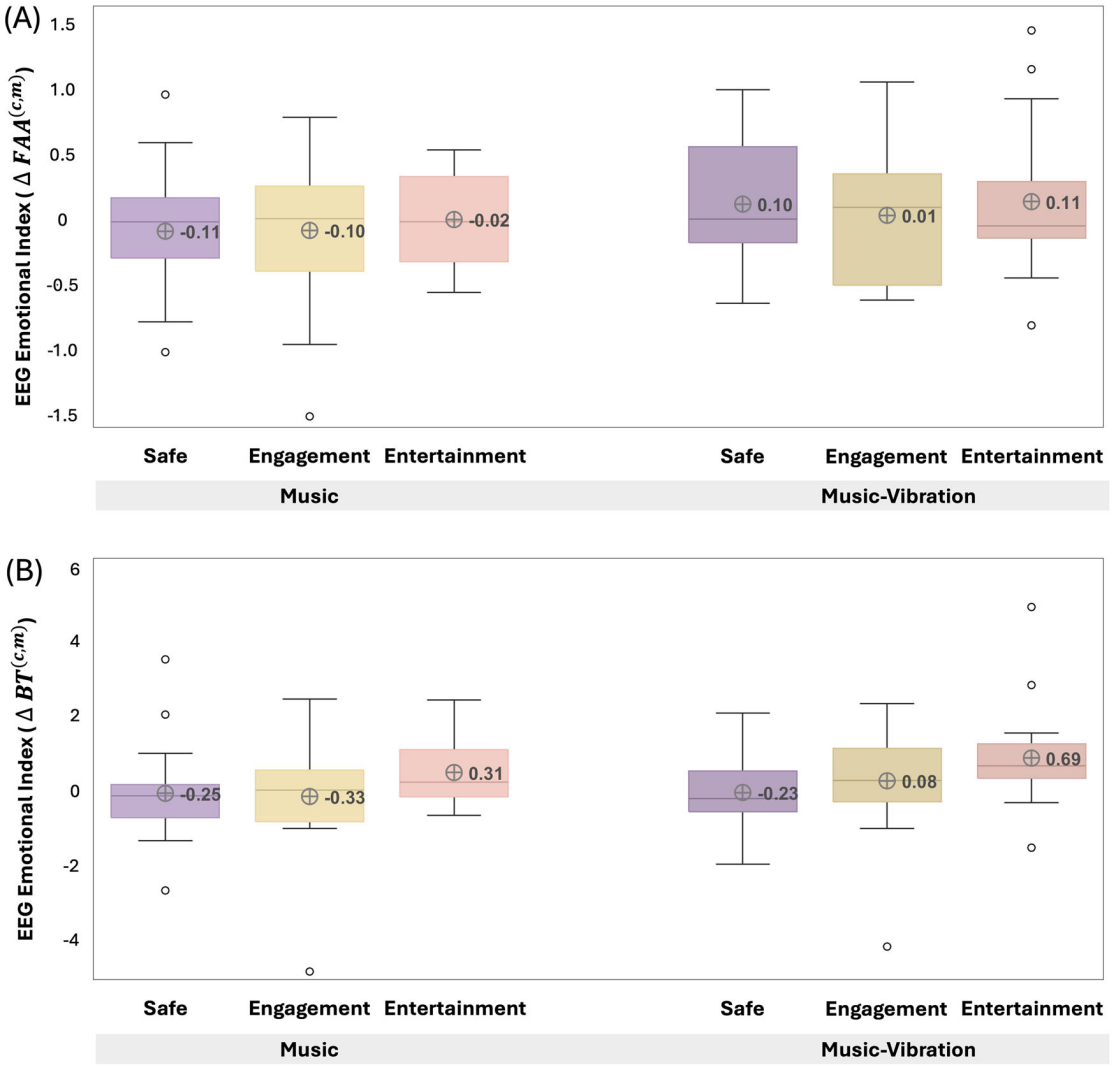
Condition‐wise comparison on pleasure, arousal, and dominance dimensions (EEG). Boxplots illustrate the distributions of EEG in (A) pleasure and (B) arousal indices across context−solution pairs (Safe, Engagement, and Entertainment), and modality (M vs. MV). Each box represents the interquartile range (IQR), the central line indicates the median, “o” denotes outliers beyond 1.5× IQR, and “⊕” represents the mean value.

Descriptive statistics are detailed by context–solution pairs and modalities in Table 2 and Figure 6. A significant main effect of modalit*y* was observed in the rmANOVA (*F*(1,17) = 5.2917, *p* = 0.0344). Post‐hoc pairwise comparisons revealed that the MV condition elicited significantly higher pleasure levels compared to the music‐only condition (Δ (M‐MV) = −0.1523, *p* = 0.0344). This finding indicates that the addition of vibration to the auditory stimulus enhanced pleasure, suggesting enhanced emotional responses under multisensory stimulation. Neither the main effect of context–solution pair nor the interaction was statistically significant (*p* > 0.05).

**TABLE 2:** Descriptive statistics of EEG—frontal alpha asymmetry (FAA) and high‐beta/theta power ratio (BT)—by context−solution pairs (Safe, Engagement, and Entertainment) and modality (M, MV).

Emotion profile		Safe		Engagement		Entertainment	
		M	M+V	M	M+V	M	M+V
FAA	Mean	−0.110	0.095	−0.105	0.010	−0.022	0.114
	Median	−0.039	−0.019	−0.015	0.070	−0.038	−0.071
	SD	0.482	0.514	0.552	0.521	0.360	0.571
	IQR	0.460	0.731	0.648	0.845	0.648	0.431
BT	Mean	−0.248	−0.226	−0.333	0.084	0.305	0.689
	Median	−0.314	−0.388	−0.165	0.096	0.050	0.484
	SD	1.309	1.040	1.512	1.486	0.818	1.336
	IQR	0.872	1.084	1.370	1.424	1.254	0.918

*Note*: M refers to music, and M+V refers to music with vibration.

#### 3.2.2 Arousal

A significant main effect of modality was also found (*F*(1,17) = 4.6165, *p* = 0.0464). Arousal index was significantly higher under the MV condition compared to the M condition (Δ (M‐MV) = −0.2744, *p* = 0.0464). These findings suggest that the combination of vibrational stimulation with auditory input significantly enhances arousal levels, highlighting the impact of multisensory integration. The main effect of context–solution pair was not statistically significant (*p* > 0.05), though post‐hoc comparisons revealed a significant difference between the Safe and Entertainment pairs, with the Entertainment pair showing higher arousal levels (Δ (M‐MV) = −0.7343, *p* = 0.0189). The interaction was not statistically significant (*p* > 0.05).

## 4 Discussion

The results supported H1: the MV condition enhanced pleasure in both behavioral and EEG measures, with arousal showing a convergent pattern in EEG (*p* < 0.05) and a marginal trend in behavioral data (*p* = 0.099) across all three context–solution pairs. Regarding H2, the behavioral data showed distinct emotional profiles across the three context–solution pairs; however, the EEG data, in which modality‐specific changes were contrasted against the video‐only baseline, did not show a significant main effect of context–solution pair. The interaction between modality and context−solution pair was not significant, indicating that the MV enhancement operated independently of the context–solution pair.

### 4.1 **Vibrotactile Enhancement Across Context**−**Solution Pairs**

The current findings showed that vibrotactile stimulation synchronized with music enhanced emotional responses compared to music alone. The main effect of modality reached significance for both pleasure and arousal in the EEG, and a convergent pattern was observed in the behavioral data (significant for pleasure, marginal for arousal). Huo et al. [106] found that tactile feedback delivered via touchscreens increased positive valence and modulated arousal based on feedback intensity and task difficulty, indicating that the design of haptic parameters can systematically affect arousal in either direction. However, much of the literature on audio−tactile integration has focused on relaxation, typically pairing vibration with sedative music or physiological signals, such as heartbeat. Brodsky [65] paired sedative music with whole‐body vibroacoustic stimulation and found enhanced subjective relaxation. Zhou et al. [107] used haptic stimulation simulating a slowed heartbeat and found reduced stress with increased comfort. Similarly, Schwartz et al. [67] demonstrated that audio−tactile music integration increased positive emotions and decreased state anxiety, with effects observed on valence rather than arousal. The present findings extend this literature and may be attributed to two design features. First, our system adopted the frequency‐band–specific, role‐based mapping. This design differs from prior studies that predominantly utilized localized, low‐frequency vibrations [67, 107]. Second, our system maintained intensity and temporal congruence between music and vibration, both of which are critical for audio−tactile integration [64]. Previous studies have reported that congruent audio‐vibrotactile stimulation enhances groove, spontaneous movement, and arousal ratings [108], and that chair‐delivered vibrotactile cues strengthen musical engagement [66]. Merchel and Altinsoy [62] similarly reported that whole‐body vibration added to music improved overall quality ratings.

Our EEG data showed no significant main effect of context−solution pair, although post‐hoc comparisons indicated Entertainment showed higher arousal than Safe, consistent with the behavioral findings. This divergence between self‐report and neurophysiological measures is consistent with the literature. Self‐report scales, such as the SAM, assess explicit, conscious aspects of emotion that integrate contextual factors and cognitive appraisal processes, whereas EEG indices assess implicit, automatic responses of emotion to sensory stimulation [109, 110]. Previous research has demonstrated that these types of measures can yield dissociable outcomes, as they engage partially distinct neural mechanisms and are differentially sensitive to various stimulus parameters [111]. In the present study, the behavioral findings may, therefore, reflect conscious appraisal integrating the driving scenarios and associated musical parameters, whereas the EEG findings reflect more automatic neural responses to the same stimuli.

The interaction between modality and context–solution pair was not statistically significant. While this finding suggests a certain robustness of audio‐vibrotactile effects across diverse in‐vehicle contexts, the use of MV across all conditions warrants a more nuanced interpretation of the descriptive data. Although the interaction was not significant, the MV condition generally increased both pleasure and arousal, and the Safe pair showed a divergent descriptive pattern. The inclusion of vibration appeared to offset the intended reduction in arousal (M Δ = −2.222; MV Δ = −1.556). This trend implies that the inherent arousal‐boosting nature of vibrotactile stimulation may run counter to the low‐arousal state prioritized for calming scenarios within the PAD framework. Conversely, in the Entertainment context, the increase in arousal (M Δ = 1.278; MV Δ = 1.889) was consistent with the target affective state. Importantly, the musical content was designed with emotion‐specific parameters such as tempo, mode, register, and dynamics to target the PAD profile of each context, whereas the vibrotactile component did not incorporate its own emotion‐specific design parameters. This asymmetry may explain why the modality effect was uniform across pairs. Future adaptive systems should, therefore, develop vibrotactile parameters that independently target the intended emotional profile, paralleling the role of musical features [106, 112, 113, 114].

### 4.2 Emotional Profiles Across Context–Solution Pairs

In the behavioral data, the three context–solution pairs exhibited distinct emotional profiles: the Safe pair showed the highest pleasure and dominance increases with the lowest arousal increase, the Entertainment pair produced the highest arousal increases, and the Engagement pair showed intermediate arousal levels. These profiles are consistent with the intended emotional targets derived from the PAD framework [25, 26] and the musical design parameters [75−76, 77, 78, 115]. As discussed in Section 4.1, these behavioral profiles reflect the integrated experience of music and driving context rather than either factor alone. Despite this limitation, the fact that each pair produced the PAD profile broadly consistent with its design target suggests that the content design methodology warrants further investigation. Below, each context–solution pair is discussed in relation to its driving scenario and musical design.

The Safe context–solution pair showed the highest pleasure and dominance increases with the lowest arousal increase among the three pairs. This profile is consistent with the PAD framework's characterization of safety [27]. The Safe pair combined a complex urban driving scenario with calm music designed for emotional recovery (70–80 BPM, tenor‐alto register, mezzo‐forte dynamics, binary form with mixed major‐minor harmony) [75, 76, 77, 78, 115]. Although EEG data showed no significant main effect of context–solution pair, the arousal pattern was descriptively consistent with behavioral findings, with lower levels in the Safe pair compared to Entertainment. The observed emotional profile is consistent with prior research demonstrating that calm, slow‐tempo music can modulate affective states toward relaxation [30, 31, 37, 38, 39], though low‐intensity music may also impair vigilance [40]. The 70–80 BPM tempo was intended to approximate resting heart rate while maintaining mezzo‐forte dynamics to ensure auditory presence without compromising alertness [41, 42, 43]. The musical design's tenor‐alto register and mixed major‐minor harmony are consistent with research identifying that middle register, slow tempo, and mode combinations are associated with peaceful emotional qualities [74, 75, 76].

The Engagement context–solution pair showed arousal higher than the Safe pair but lower than Entertainment, with pleasure and dominance increases comparable to Entertainment and significantly lower than Safe. Within the PAD framework, engagement is characterized by moderate arousal and moderate‐to‐high dominance, maintaining optimal alertness without cognitive overload [28]. The observed profile partially matches this characterization, with moderate arousal and positive emotional states. This pair combined a highway cruising scenario with music designed for sustained alertness. The cruising scenario simulated sustained driving at approximately 100 km/h, a monotonous environment prone to drowsiness and disengagement [116, 117]. The music (60–70 BPM, tenor‐soprano register, piano to mezzo‐forte dynamics) was intended to maintain driver alertness without inducing overarousal. The moderate arousal observed may reflect both the musical stimulation and the low‐variability driving context. This tempo is consistent with Li et al. [117], who found that medium‐tempo music (60–90 BPM) was most effective at reducing fatigue during monotonous highway driving. This design is particularly relevant for SAE Level 3 automation, where occupants must remain available for takeover while engaging in nondriving activities [49].

The Entertainment context–solution pair produced the highest arousal increases, with EEG post‐hoc comparisons also showing higher arousal than the Safe pair. The observed profile partially matches the PAD framework's characterization of entertainment [29], as the highest arousal was achieved, but pleasure did not reach the levels observed in the Safe pair. This pair combined a highway entry scenario with energizing music (112–122 BPM, soprano register, mezzo‐forte to forte dynamics). The highway entry scenario involved acceleration and merging maneuvers that naturally elicit moderate‐to‐high arousal. The music was intended to amplify the excitement associated with this dynamic driving context. The high arousal observed is consistent with prior research showing that fast‐tempo music is associated with excitement and heightened arousal [118, 119, 120], though the dynamic driving scenario likely also contributed to the elevated arousal levels. The attenuated pleasure response compared to the Safe pair may reflect the more stressful nature of the highway entry scenario. Even without active driving tasks, the perception of acceleration and merging into the highway probably limited the increase in pleasure. This indicates that for high automation (SAE Level 4 and above), multisensory systems should be designed to balance high arousal with passenger comfort during dynamic vehicle movements [121].

## 5 Conclusion

This study developed contextually adaptive music‐based multisensory content for autonomous driving environments and evaluated whether the addition of synchronized vibrotactile stimulation enhances emotional responses compared to music alone. The MV condition enhanced pleasure compared to M in both self‐reported and neurophysiological measures, with arousal showing a similar pattern (H1 supported). This enhancement was consistent across the three context–solution pairs examined. Distinct emotional profiles were also observed across the context–solution pairs in the behavioral data (H2). These differences, however, cannot be attributed to the musical intervention or the driving scenario alone, because each musical solution was paired with a unique driving scenario and because the EEG data did not show a significant context–solution pair effect after controlling for the video‐only baseline.

This study has several limitations. First, the relatively small sample size may have affected the generalizability of the results. Second, the neurophysiological analysis included only pleasure and arousal because EEG indices for the dominance dimension are not yet well‐established. Third, each musical solution was paired with a single driving scenario, and behavioral measures were not collected during the video‐only (without solution) section. As a result, the contributions of music and driving scenarios to the behavioral pair differences cannot be separated. The modality comparison itself remained valid because identical driving video content was used in both M and MV conditions within each pair. This design prioritized ecological validity, but future studies should employ factorial designs that independently manipulate music and driving scenarios, with behavioral measures collected across all conditions. Fourth, we did not include a vibration‐only condition because our theoretical framework conceptualized vibration as an enhancement modality rather than a standalone intervention, and vibrotactile stimulation without accompanying music lacks ecological validity for in‐vehicle applications. Lastly, the use of uniform vibration parameters across all context–solution pairs may have been suboptimal, particularly for the Safe context, where arousal reduction was desired.

Despite these limitations, this study demonstrates that vibrotactile enhancement of music provides emotional benefits in simulated autonomous driving, as evidenced by converging behavioral and neurophysiological measures. The consistency of this effect across the three context–solution pairs examined suggests its practical potential for in‐vehicle experience design. The systematic development of context‐specific multisensory content based on the PAD framework provides a grounded design methodology. Combining SAM with EEG allowed us to track both conscious appraisal and implicit neural responses within a single session. We identify several future directions for extending this research. First, studies with larger and more diverse samples would enhance statistical power and generalizability. Second, developing and validating EEG‐based dominance indices would enable a comprehensive neurophysiological assessment of all three PAD dimensions. Third, future factorial designs that include video‐only and vibration‐only conditions would systematically decompose the contributions of auditory, tactile, and combined multisensory stimulation. Fourth, future work should develop adaptive vibrotactile systems that modulate parameters according to the target emotional state. As discussed in Section 4.1, such context‐specific calibration could preserve the pleasure‐enhancing benefits of vibration while ensuring arousal modulation remains congruent with the intended affective profile. Further, given that the emotional effectiveness of vibrotactile cues is highly context‐dependent and individual differences in vibration sensitivity exist, investigating personalized vibration profiles will be essential to optimize multisensory interventions for diverse autonomous driving environments.

## Author Contributions

E.J.: Conceptualization, methodology, formal analysis, writing – original draft, writing – review and editing; Y.J.H.: Data curation, formal analysis, writing – original draft; J.S.: Methodology, investigation, writing – original draft; J.S.K.: Investigation, data curation, formal analysis, writing – original draft; M.A.Y.: Formal analysis, data curation, formal analysis, writing – original draft; S.‐P.K.: Conceptualization, formal analysis, writing – original draft, writing – review and editing. All authors have read and agreed to the published version of the manuscript.

## Conflicts of Interest

The authors declare that there is no conflict of interest regarding the publication of this study.

## Supporting information




**Supplementary Table**: nyas70305‐sup‐0001‐TableS1.docx
