# Supplementary material for: Psychological Empowerment on the Streets: Designing and Validating Multisensory Experiences in Simulated Autonomous Driving
Source: Ann N Y Acad Sci. 2026 Jun 16;1560(1):e70305. doi: 10.1111/nyas.70305 (PMC13271048; doi:10.1111/nyas.70305)
Supplement: Supplementary file 1 — Supplementary Table: nyas70305‐sup‐0001‐TableS1.docx [file NYAS-1560-0-s001.docx]

Table S1. Overview of driving scenarios, phases, and solutions

| Phase | Video | Measures | Set1 | Set2 | Set3 | Time (sec) |
| --- | --- | --- | --- | --- | --- | --- |
| Departure | Parking to urban roads | Baseline |  |  |  | 60 |
| City driving | Urban driving with stress events |  |  |  |  | 60 |
|  | Stable urban driving | Pre1 |  |  |  | 60 |
|  |  |  | VO | Safe solution in MV | Safe solution in M | 90 |
|  |  | Post1 |  |  |  | 60 |
| Highway entry | Highway entry and acceleration |  |  |  |  | 60 |
|  | Highway driving at stable speed | Pre2 |  |  |  | 60 |
|  |  |  | Entertainment solution in M | VO | Entertainment solution in MV | 90 |
|  |  | Post2 |  |  |  | 60 |
| Highway cruising | Takeover and highway cruising |  |  |  |  | 60 |
|  | Highway cruising | Pre3 |  |  |  | 60 |
|  |  |  | Engagement solution in MV | Engagement solution in M | VO | 90 |
| Arrival | Returning | Post3 |  |  |  | 60 |

VO: video watching only with no solution, MV: music with vibration, M: music only. EEG was measured through all phases.
